# The role of the VEGF-C/VEGFR-3 axis in cancer progression

**DOI:** 10.1038/sj.bjc.6603487

**Published:** 2006-12-12

**Authors:** J-L Su, C-J Yen, P-S Chen, S-E Chuang, C-C Hong, I-H Kuo, H-Y Chen, M-C Hung, M-L Kuo

**Affiliations:** 1 Institute of Medical Science, College of Medicine, China Medical University, Taichung 404, Taiwan; 2 Center for Molecular Medical, China Medical University Hospital, Taichung 404, Taiwan; 3 Department of Biotechnology and Bioinformatics, Asia University, Taichung 41354, Taiwan; 4 Department of Molecular and Cellular Oncology, The University of Texas MD Anderson Cancer Center, Houston, TX 77030, USA; 5 Department of Internal Medicine, National Cheng Kung University Hospital, Tainan 70428, Taiwan; 6 Institute of Clinical Medicine, National Cheng Kung University Hospital, Tainan 70428, Taiwan; 7 Institute of Toxicology, College of Medicine, National Taiwan University, Taipei 100, Taiwan; 8 Division of Cancer Research, National Health Research Institutes, Taipei 10016, Taiwan; 9 Graduate School of Biomedical Sciences, The University of Texas Health Science Center at Houston, Houston, TX 77030, USA; 10 Angiogenesis Research Center, National Taiwan University, Taipei, Taiwan

**Keywords:** VEGF-C, VEGFR-3, lymphangiogenesis, metastasis

## Abstract

Vascular endothelial growth factor (VEGF) receptor 3 (VEGFR-3) (also called VEGFR-3) is activated by its specific ligand, VEGF-C, which promotes cancer progression. The VEGF-C/VEGFR-3 axis is expressed not only by lymphatic endothelial cells but also by a variety of human tumour cells. Activation of the VEGF-C/VEGFR-3 axis in lymphatic endothelial cells can facilitate metastasis by increasing the formation of lymphatic vessels (lymphangiogenesis) within and around tumours. The VEGF-C/VEGFR-3 axis plays a critical role in leukaemic cell proliferation, survival, and resistance to chemotherapy. Moreover, activation of the VEGF-C/VEGFR-3 axis in several types of solid tumours enhances cancer cell mobility and invasion capabilities, promoting cancer cell metastasis. In this review, we discuss the novel function and molecular mechanism of the VEGF-C/VEGFR-3 axis in cancer progression.

Tumour invasion and metastasis are the critical steps in determining the aggressive phenotype of human cancers and the principal cause of cancer deaths. A better understanding of the molecular events that lead to metastasis and of the complex interactions between metastatic cells and host factors is essential for designing more effective cancer therapies. One of these events is the endogenous production of growth factors that act on tumours via functional external receptors.

The vascular endothelial growth factor (*VEGF*) gene family, which encodes five polypeptide growth factors, VEGF-A, -B, -C, -D, and -E, is particularly important because of its angiogenic and lymphangiogenic properties that promote the growth and metastasis of neoplasms (Ferrara and Davis-Smyth, 1997). Vascular endothelial growth factors achieve this by activating related tyrosine kinase receptors termed VEGFR-1 (Flt-1) and VEGFR-2 (KDR). These receptors are mainly expressed by endothelial cells (Ferrara and Davis-Smyth, 1997) but are also expressed by a wide variety of cancer cell lines (Liu *et al*, 1995; Strizzi *et al*, 2001; Wu *et al*, 2003). The expression of both VEGF receptors (VEGFRs) and their ligands, results in an autocrine function. For example, in melanoma (Liu *et al*, 1995) and mesothelioma (Strizzi *et al*, 2001) cells, exogenous VEGF-A stimulates cell proliferation by activating VEGFR-2. Vascular endothelial growth factor-A also stimulates human leukaemic cell proliferation, migration, and matrix metalloproteinase 9 production through VEGFR-2 (Dias *et al*, 2002). In addition, VEGF-A induces the activation of mitogen-activated protein kinases (MAPKs), promotes cell growth in human pancreatic and breast cancer cells (von Marschall *et al*, 2000), and promotes cell adhesion and migration through activated integrin *α*v*β*3 in M21 melanoma cells (Byzova *et al*, 2000). These reports highlight the importance of VEGFs/VEGFRs signaling in the biology of a variety of tumour cells and illustrate its well-described role.

Vascular endothelial growth factor-C has been characterised as a lymphangiogenic and angiogenic growth factor and has been shown to signal through the receptors VEGFR-3 (also called Flt-4) and VEGFR-2 (Plate, 2001). Vascular endothelial growth factor receptor 3 has also been shown to be important in determining the potential for a lymphangiogenic response. Recent studies have indicated that VEGFR-3, which has been proposed as a marker for lymphatic endothelial cells, is also expressed in a variety of human malignancies. Vascular endothelial growth factor receptor 3 expression in colon cancer has been associated with poorer survival, suggesting an axis between VEGF-C and VEGFR-3 in colorectal cancer (Witte *et al*, 2002). Expression of VEGFR-3 has also been reported to be significantly correlated with the different stages of cervical carcinogenesis (Van Trappen *et al*, 2003). The VEGF-C/VEGFR-3 axis exerts different biological effects on cancer cells to cause tumour progression. For example, in our previous study (Su *et al*, 2006), we provided evidence that the VEGF-C/VEGFR-3 axis enhances cancer cell mobility and invasion capabilities and promotes cancer cell metastasis.

We concluded from these various observations that, the VEGF-C/VEGFR-3 axis, through different signaling pathways, plays a critical role in cancer progression by regulating different cellular functions, such as invasion, proliferation, and resistance to chemotherapy.

## THE VEGF-C/VEGFR-3 AXIS AFFECTS TUMOUR PROGRESSION BY REGULATING LYMPHANGIOGENESIS

Lymphangiogenesis and sustained angiogenesis are important steps in tumour progression. Similar to angiogenesis, a tumour can induce its own network of lymphatics that connect with the surrounding lymphatic vessels. However, clinical and pathological observations suggest that for many carcinomas, the transport of tumour cells by lymphatics is the most common pathway of initial dissemination, with cancer spread by afferent lymphatics following routes of natural drainage. Until recently, tumour-induced lymphangiogenesis was a relatively unfamiliar concept in the field of cancer research.

One could envisage that overproduction of lymphangiogenic factors may provide extra lymphatic channels, thus promoting the spread of tumour cells. It has been reported that two members of the VEGF family, VEGF-C and VEGF-D, not only are important regulators of lymphangiogenesis *in vivo*, but also enhance lymphatic metastasis (Karpanen and Alitalo, 2001; Skobe *et al*, 2001). Previous studies have demonstrated that VEGF-C is the lymphangiogenic factor that induces the formation of lymphatic vessels within and around tumours. In addition, these studies have shown that VEGF-C-overexpressing tumours increase intratumoral lymphangiogenesis by activating the VEGF-C/VEGFR-3 axis in lymphatic endothelial cells, enhancing metastatic spread via the lymphatics (Karpanen and Alitalo, 2001; Skobe *et al*, 2001). These discoveries affect how we currently think about tumours; that is, the spread of cancer cells from the primary tumour to the lymphatics and blood stream, which has been previously attributed to the general invasive properties of the tumour, is now understood to be due to active recruitment of new lymphatics by tumour-derived VEGF-C or VEGF-D (Karpanen and Alitalo, 2001; Plate, 2001; Karkkainen *et al*, 2002).

In animal models, the induction of lymphangiogenesis by the VEGF-C/VEGFR-3 axis increased tumour metastasis via the lymphatic system (Plate, 2001; Skobe *et al*, 2001). Vascular endothelial growth factor-C has been detected in many human cancers, and a number of reports have shown a correlation between VEGF-C expression in human tumours and the formation of metastases in regional lymph nodes. So far, the VEGF-C levels in several types of primary tumours have correlated significantly with lymph node metastasis (Akagi *et al*, 2000; Hashimoto *et al*, 2001; Witte *et al*, 2002; Juttner *et al*, 2006; Su *et al*, 2006). However, there are also some reports did not find a correlation between VEGF-C expression and metastasis (Gunningham *et al*, 2000; George *et al*, 2001; Komuro *et al*, 2001; Hanrahan *et al*, 2003; Koyama *et al*, 2003).

VEGF-C overexpressing tumours not only increase intratumoral lymphangiogenesis but also the peri-tumoral amount of lymphatic vessels. There are several papers report that peri-tumoral, but not intratumoral, lymphatic vessels were shown to be functional and it was propose that lymphatic vessels at the tumour margin may play a major role in lymphatic spread of tumour cells (Padera *et al*, 2002; Gombos *et al*, 2005). An important point in the field of lymphangiogenesis is to find out whether intratumour lymphatic vessels are functional for tumour dissemination into the lymph node.

## THE ROLE OF THE VEGF-C/VEGFR-3 AXIS IN TUMOUR CELLS

Vascular endothelial growth factor receptors are primarily expressed by endothelial cells, but are also expressed by nonendothelial cell types, including several cancer cells (Weninger *et al*, 1999; Yonemura *et al*, 2001; Witte *et al*, 2002; Li *et al*, 2003; Neuchrist *et al*, 2003; Van Trappen *et al*, 2003; Wu *et al*, 2003). Vascular endothelial growth factors play a critical role in pathological conditions by binding to and activating VEGFRs. Many tumour cells and tumour-associated vasculature express both VEGFRs and VEGFs, and an autocrine/paracrine function has been proposed (Byzova *et al*, 2000; Dias *et al*, 2002).

Although expression of VEGF-C and VEGFR-3 has been significantly and negatively correlated to the progression of certain selective types of cancer (Table 1), the function of the VEGF-C/VEGFR-3 axis in cancer cells is largely unknown. Vascular endothelial growth factor receptor 3 expression in colon cancer, which as noted earlier has been associated with poorer survival (Witte *et al*, 2002) and has been significantly correlated with the different stages of cervical carcinogenesis (Van Trappen *et al*, 2003), suggesting an axis between VEGF-C and VEGFR-3 in cancer cells. However, there are some studies that did not detect VEGFR-3 expression by tumour cells (Chen *et al*, 2004; Zeng *et al*, 2004) and these controversial results might be clarified by more powerful and sensitive technique in the future.

Immunohistochemical staining has shown that VEGFR-3 is expressed by Kaposi sarcoma cells (Weninger *et al*, 1999). Furthermore, Marchio *et al* (1999) reported that tyrosine phosphorylation of VEGFR-3 was increased in Kaposi sarcoma cells treated with VEGF-C recombinant protein as well as C156S mutant VEGF-C recombinant protein, a selective ligand and an activator of VEGFR-3, although devoid of any Flt-1 activation property. Marchio *et al* (1999) found that the activation of the VEGF-C/VEGFR-3 axis in Kaposi sarcoma cells was indeed involved in the regulation of cellular functions, such as proliferation and migration. Using endothelial cells as a control, Marchio *et al* (1999) further found that the activation of the VEGF-C/VEGFR-3 axis significantly increased the proliferation and migration of KS IMM Kaposi sarcoma cells in a dose-dependent manner.

The VEGF-C/VEGFR-3 axis has also been found to play a role in the growth of malignant mesothelioma cells (Masood *et al*, 2003). Indeed, both the VEGF-A/VEGFR-2 and VEGF-C/VEGFR-3 axes are present in several malignant pleural mesothelioma cell lines, and both axes are functionally related to cell growth. Further supporting the role of the VEGF-C/VEGFR-3 axis in cell growth was the finding that using VEGF-C antisense oligonucleotides, recombinant VEGFR-3/Fc, or VEGFR-3 antibody to inhibit the activity of the VEGF-C/VEGFR-3 axis in malignant pleural mesothelioma cells resulted in a significant reduction in cell viability. However, there are now many studies, including our previous study, demonstrate that VEGF-C overexpression by experimental tumours did not enhance tumour growth but selectively promote tumour metastasis (Skobe *et al*, 2001; Hoshida *et al*, 2006; Su *et al*, 2006). In addition, other studies have been reported that neither expression of VEGFR-3-Ig in LNM35 lung cancer cells (He *et al*, 2002) nor expression of VEGF-C-specific small interfering RNA vector in C166 mammary tumour cells (Chen *et al*, 2005) changed the tumour growth rate.

The interaction of VEGF-C with VEGFR-3 in leukaemia cells promotes cell survival and proliferation, as shown by Dias *et al* (2002) in two cell lines and in five cases of VEGFR-3^+^ primary leukaemia. In particular, these researchers observed that VEGF-C and a mutant form of the molecule that lacks the KDR-binding motif induced receptor phosphorylation and increased cell proliferation and survival, as shown by increased Bcl-2/Bax ratios. Moreover, the activation of the VEGF-C/VEGFR-3 axis protected leukaemic cells from the apoptotic effects of chemotherapeutic agents such as cytarabine, daunorubicin, and etoposide. These results also identified the VEGF-C/VEGFR-3 pathway as a novel therapeutic target for the treatment of subsets of acute leukaemia.

Our recent study (Su *et al*, 2006) provided evidence that the VEGF-C/VEGFR-3 axis enhances cancer cell mobility and invasiveness and contributes to the promotion of cancer cell metastasis in various types of cancer, including lung adenocarcinoma, breast cancer, cervical prostate cancer, and colorectal cancer. Further, the VEGF-C/VEGFR-3-mediated invasion and metastasis of cancer cells was found to require upregulation of the neural cell adhesion molecule contactin-1 through activation of the Src-p38 MAPK-C/EBP-dependent pathway. In agreement with the role of the VEGF-C/VEGFR-3 axis in invasion and metastasis, an examination of tumour tissues from various types of cancers revealed high levels of VEGFR-3 and VEGF-C expression that correlated closely with clinical metastasis and patient survival. The function and molecular mechanism of the VEGF-C/VEGFR-3 axis were revealed from *in vitro* and *in vivo* studies and in an examination of patient outcomes.

## CONCLUSIONS

The VEGF-C/VEGFR-3 axis plays a critical role in cancer progression by inducing lymphangiogenesis and facilitating the mobility of several types of cancer cells (Su *et al*, 2006). The VEGF-C/VEGFR-3 axis may affect cancer development or progression by directly affecting tumour cells. Unlike the well-characterised axis of VEGF-A and VEGFR-2, there may be many undefined functions and molecular mechanisms involved in the tumour progression mediated by the VEGF-C/VEGFR-3 axis; thus, further study of the axis is needed. It is also important to identify the functions of the VEGF-C/VEGFR-3 axis in macrophage infiltration/activation and to determine how these tumour-educated macrophages affect tumour progression. This is because infiltrating macrophages have correlated significantly and negatively with cancer patient prognosis and survival (Ogawa *et al*, 2004; Schoppmann *et al*, 2006). It has been reported that tumour-associated macrophages play a crucial role in angiogenesis and lymphangiogenesis under inflammatory condition (Cursiefen *et al*, 2004; Maruyama *et al*, 2005). It also has been reported that macrophages support lymphangiogenesis in two different ways, either by transdifferentiating and directly incorporating into the endothelial layer or by stimulating division of pre-existent local lymphatic endothelial cells (Kerjaschki, 2005; Maruyama *et al*, 2005). In our unpublished data, we also found that VEGF-C plays a critical role in the macrophage infiltration in lung tumour through VEGFR-3 and this VEGFR-3-mediated macrophage infiltration may involved in radiosensitisation of lung cancer cells.

Another important area to be further explored is the nuclear localisation of VEGFR-3 and other VEGF receptors. This is because many receptor tyrosine kinases are detected in the cell nucleus and function as transcription cofactors to activate gene promoters, such as the EGFR family (Lo and Hung, 2006). Nuclear translocation of EGFRs has been reported to play a critical role in cancer progression (Lo and Hung, 2006). We have found that VEGF-C activated VEGFR-3 was translocated into the nucleus of both lung adenocarcinoma cells and primary lymphatic endothelial cells (our unpublished data). Other aspects of the physical functions and molecular mechanisms of this nuclear localisation remain unclear and need to be clarified.

Targeting the VEGF-C/VEGFR-3 axis may be therapeutically significant for certain types of tumours. Thus, the continued discovery and characterisation of factors that regulate VEGF-C or VEGFR-3 will be essential for developing new therapies that limit the spread of cancer. In particular, new drugs that block the VEGF-C/VEGFR-3 signaling pathway may provide useful anticancer therapeutics by mechanisms other than the blockage of lymphangiogenesis. In addition to being potential targets for inhibiting tumour metastasis, factors implicated in tumour lymphangiogenesis and the specific molecules found on the activated lymphatic endothelium may prove valuable in the diagnosis of particularly aggressive metastatic cancers.

## Figures and Tables

**Table 1:** Expression, function, and clinical significance of VEGFR-3 in human tumours

**Tumo**u**r type**	**Primary findings**	**References**
Gastric cancer	The expression of VEGFR-3 correlated with reduced carcinoma-specific survival, and a Cox multivariate regression analysis qualified VEGFR-3 as an independent prognostic parameter. The presence of the VEGF-C/VEGFR-3 axis was associated with poor survival in lymph node-positive gastric cancer	Juttner *et al* (2006)
		
Breast cancer	The expression of VEGFR-3 in breast cancer tissue was not significantly related to tumour grade (*P*=0.063)	Longatto Filho *et al* (2005)
	The activation of the VEGF-C/VEGFR-3 axis increased cell mobility of breast cancer cells. The expression of VEGFR-3 in breast tumour tissue was higher than it was in matched normal tissue	Su *et al* (2006)
		
NSCLC	VEGF-C and VEGFR-3 status may be indicative of survival rates for patients with T1 lung adenocarcinoma	Kojima *et al* (2005)
	VEGF-C and VEGFR-3 expression may be indicative of survival rates for patients with NSCLC	Arinaga *et al* (2003)
	In NSCLC, the VEGF-C/VEGFR-3 axis is related to lymphangiogenesis, and angiogenesis and to the occurrence and development of lung cancers. VEGF-C expression could be a useful predictor of poor prognosis in NSCLC	Li *et al* (2003)
	The VEGF-C/VEGFR-3 axis enhances cancer cell mobility and invasiveness and contributes to the promotion of cancer cell metastasis Examination of tumour tissues from various types of cancers revealed high levels of VEGFR-3 and VEGF-C expression that correlated closely with clinical metastasis and patient survival	Su *et al* (2006)
		
Leukaemia	The VEGF-C/VEGFR-3 axis mediates leukaemic cell proliferation, survival, and resistance to chemotherapy The VEGF-C/VEGFR-3 pathway is a novel therapeutic target for the treatment of subsets of acute leukaemia	Dias *et al* (2002)
		
Cervical cancer	A significant positive correlation was found between VEGF-C and VEGFR-3 expression through the different stages of cervical carcinogenesis. These findings suggest an autocrine growth stimulation pattern via VEGFR-3 in cervical carcinoma cells	Van Trappen *et al* (2003)
		
Colorectal cancer	The expression of VEGFR-3 in >25% of the cancer cells was associated with significantly poorer overall survival (*P*<0.05), but not with lymph node metastasis or depth of tumour invasion These results suggest that VEGFs promote cancer growth not only by stimulating angiogenesis, but also by acting on receptors present on the cancer cells themselves	Witte *et al* (2002)
	The expression of VEGFR-3 in colorectal tumour tissue was higher than it was in matched normal tissue	Su *et al* (2006)
		
Prostate cancer	Significantly upregulated expressions of VEGF-A, VEGF-C, and VEGFR-3 were all found in malignant epithelium/cancer cells compared with adjacent benign epithelium (*P*<0.01) Patients in stage D had a significantly higher score than did patients in stage A, B, or C when comparing the expression of VEGF-C or VEGFR-3 in the tumour area (*P*<0.01)	Yang *et al* (2006)
	The increased expression of the VEGF-C /VEGFR-3 axis played a role in prostate cancer progression and in metastasis to regional lymph nodes	Jennbacken *et al* (2005)
	VEGFR-3 expression was associated with tumour progression and may play an important role in facilitating the lymphatic spread of prostate carcinomas; a high level of VEGFR-3 in prostate cancer cells increases the risk of biochemical recurrence in prostate cancer patients treated by radical prostatectomy	Li *et al* (2004)
		
Kaposi sarcoma	The VEGF-C/VEGFR-3 axis stimulates the migration and proliferation of Kaposi sarcoma cells	Marchio *et al* (1999)
		
Head and neck squamous cell carcinoma	The broad expression of the VEGF-C/VEGFR-3 axis in head and neck squamous cell carcinoma suggests involvement in tumour lymphangiogenesis and angiogenesis, promoting tumour growth, and propagation of cancer cells. This implies that inhibitors of lymphangiogenesis could become effective therapeutic options	Neuchrist *et al* (2003)
		
Endometrial carcinoma	The presence of VEGF-D and VEGFR-3 in endometrial carcinoma may predict myometrial invasion and lymph node metastasis and may prospectively identify patients who are at increased risk for poor outcome. In addition, VEGF-D and VEGFR-3 may be promising targets for new therapeutic strategies in endometrial carcinoma	Yokoyama *et al* (2003)
		
Mesothelioma	VEGF/Flt-1 and VEGF-C/VEGFR-3 autocrine loops targeting agents significantly inhibited malignant mesothelioma cell growth	Masood *et al* (2003)

NSCLC=non-small cell lung cancer; VEGF=vascular endothelial growth factor; VEGFR=VEGF receptor.
